# HMGB1 mediates cognitive impairment caused by the NLRP3 inflammasome in the late stage of traumatic brain injury

**DOI:** 10.1186/s12974-021-02274-0

**Published:** 2021-10-19

**Authors:** Si-Wei Tan, Yan Zhao, Ping Li, Ya-Lei Ning, Zhi-Zhong Huang, Nan Yang, Dong Liu, Yuan-Guo Zhou

**Affiliations:** 1 State Key Laboratory of Trauma, Burns and Combined Injury, Department of Occupational Disease, Daping Hospital, Army Medical University, Chongqing, China; 2 Medical Center of Trauma and War Injury, State Key Laboratory of Trauma, Burns and Combined Injury, Daping Hospital, Army Medical University, Chongqing, China

**Keywords:** NLRP3 inflammasome, HMGB1, Cognitive dysfunction, Traumatic brain injury

## Abstract

**Background:**

Cognitive impairment in the late stage of traumatic brain injury (TBI) is associated with the NOD-, LRR and pyrin domain-containing protein 3 (NLRP3) inflammasome, which plays an important role in neuroinflammation. Although classical inflammatory pathways have been well-documented in the late stage of TBI (4–8 weeks post-injury), the mechanism by which the NLRP3 inflammasome impairs cognition is still unclear.

**Methods:**

Mice lacking the gene encoding for NLRP3 (NLRP3-knockout mice) and their wild-type littermates were used in a controlled cortical impact model of TBI. Levels of NLRP3 inflammasome and inflammatory factors such as IL-1β and HMGB1 were detected in post-injury hippocampal tissue, as well as long-term potentiation. Behaviors were assessed by T-maze test, novel object recognition, and nesting tests. Glycyrrhizin was used to antagonize HMGB1. Calcium imaging were performed on primary neuronal cultures.

**Results:**

By using the NLRP3-knockout TBI model, we found that the continuous activation of the NLRP3 inflammasome and high mobility group box 1 (HMGB1) release were closely related to cognitive impairment. We also found that inhibition of HMGB1 improved LTP reduction and cognitive function by increasing the phosphorylation level of the NMDAR1 subunit at serine 896 while reducing NLRP3 inflammasome activation.

**Conclusion:**

NLRP3 inflammasome damages memory in the late stage of TBI primarily through HMGB1 upregulation and provides an explanation for the long-term progression of cognitive dysfunction.

**Supplementary Information:**

The online version contains supplementary material available at 10.1186/s12974-021-02274-0.

## Background

Traumatic brain injury (TBI) is not only a common cause of acute lethality, but often progresses to cognitive disorder in the late stage [1]. The clinical manifestations are usually short-term memory loss and cognitive impairment [2]. Although current studies based on a large number of autopsies indicate that the prefrontal cortex (PFC) and hippocampus are the brain regions susceptible to TBI [3], the specific mechanism by which short-term memory impairment occurs in the late stage of TBI remains unclear. Neuroinflammation in the PFC and hippocampus is not only the pathophysiological mechanism underlying late-stage TBI but also an important pathogenic process that causes cognitive impairment progression [4] in the late stage of TBI.

The neuroinflammation caused by the NOD-, LRR and pyrin domain-containing protein 3 (NLRP3) inflammasome and its role in cognitive impairment are receiving increasing attention. For example, in the pathogenesis of Alzheimer's disease (AD), the NLRP3 inflammasome can not only damage cognition through interleukin-1β (IL-1β), which is the classic inflammatory pathway, but can also reduce Aβ removal efficiency and cause spatial memory impairment [5, 6]. NLRP3 is proposed to be a new TBI biomarker because its levels in the cerebrospinal fluid of patients 6 months after TBI injury negatively correlate with the Glasgow Outcome Scale [7]. TBI has been well documented to promote both priming and activation of the NLRP3 inflammasome through several factors [8], while the role of the NLRP3 inflammasome in the late phase of TBI (for mice > 4 w), especially in the development of TBI-induced cognitive impairment, remains unclear. One of the main issues is that inflammatory factors, such as IL-1β and IL-18, tend to return to baseline levels within 3 days of TBI [9], but the formation and development of cognitive impairment and neurodegeneration continue.

High mobility group box 1 (HMGB1) is another effector molecule induced by NLRP3 inflammasome activation and is also a typical damage-associated molecular pattern (DAMP) that participates in the priming process of the NLRP3 inflammasome [10]. In the brain, HMGB1 is often actively released by microglia, and this release is associated with the NLRP3 inflammasome [11]. A number of studies have examined the links between HMGB1 and TBI [12–14], with one notable finding being that high levels of HMGB1 can still be detected in the brain tissue of TBI patients 20 days after injury [14], suggesting that HMGB1 participates in the development of cognitive impairment in the late phase of TBI.

In this study, we studied a late-stage TBI model (4–8 weeks) with NLRP3-knockout (KO) and wild-type (WT) mice and conducted behavioral tests, field excitatory postsynaptic potential (fEPSP) recordings and molecular biology analyses to confirm that the continuous activation of the NLRP3 inflammasome and elevated HMGB1 are the key causes of TBI-induced cognitive impairment.

## Methods

### Animals

C57BL6 mice (male, weighing 22–26 g, 3 months old) were purchased and housed in a sterile environment with controlled temperature and humidity (SPF grade) at the animal center of Daping Hospital, Army Medical University. NLRP3-knockout mice were generated by homologous recombination in embryonic stem cells by replacing exons I and II of the cryopyrin/Cias1 gene (encoding the amino-terminal Pyrin domain) with an IRES-β-gal-neomycin resistance cassette using a targeting vector. A positive embryonic stem cell clone was used to generate chimeric mice. 129/C57BL/6 chimeric mice were crossed with C57BL/6 females to generate heterozygous mice [15]. NLRP3-knockout mice backcrossed to C57BL/6 J mice for 8 generations were obtained from Army Medical University. All animal experiments were performed according to the ARRIVE guidelines and received ethical approval from the Army Medical University.

The animal sample size (significance level, *α* = 0.05; power, 1−*β* = 80%; effect size, Cohen’s *d* > 0.8) was determined in the online calculating website http://powerandsamplesize.com/, according to the data of our preliminary behavioral tests. Besides, total number of mice used in the experiment (Additional file 1: Table S1) and a detailed sample size calculation table (Additional file 1: Table S2) are attached in the Supplementary materials. Mice were allocated to different experimental groups according to random number table. According to previous reports [20] and our preliminary experiments, the mortality, and dropouts of standard moderate TBI is zero.

### Traumatic brain injury model

The controlled cortical impact method was performed as described previously to generate a moderate TBI model [16]. Briefly, the mice were anesthetized with an intraperitoneal injection of 50 mg/kg pentobarbital sodium and subjected to a 5-mm diameter craniotomy using a motorized drill over the left parietal cortex. We produced a controlled cortical impact with an aerodynamic impact device (brain injury device TBI-0310, PSI, USA) using a metal tip with a 3-mm diameter. An impact location 2 mm below the dura, a 100 ms dwell time and a 3.5 m/s impact speed were set to generate the moderate TBI model. We used an electric heating blanket to maintain the body temperature of the mice. Thickened bedding material was prepared to facilitate food and water intake. In the Sham group, the mice did not suffer the impact, while the other operations, including surgical procedures (anesthesia and craniotomy), were the same as those in the TBI group. The neurologic deficit score and brain edema were measured 24 h after TBI (data not shown) as we descried previously [17] to ensure that the present model met our previous standards of moderate TBI. Briefly, neuromuscular function (forelimb flexion, torso twisting, lateral push, hindlimb placement, forelimb placement, inclined board, mobility), vestibulomotor function (balance beam) and complex neuromotor function (beam walk) were evaluated and scored with values of 0–1 or 0–2 in neuromuscular function, 0–6 in vestibulum function and 0–5 in complex neuromotor function. The total neurologic deficit score was equal to the summation of all scoring values. After the measurement of neurological deficits, the mice were sacrificed and the injured hemispheric cortex tissues between the bregma and lambdoid suture were dissected out to assay the brain water content by a wet–dry method as described previously [18].

### Immunofluorescence staining

Following TBI, the mice were perfused with ice-cold 4% paraformaldehyde in PBS (pH 7.4). The brains were post-fixed in 4% paraformaldehyde for 24 h, and coronal sections were cut serially at 4-μm thickness for paraffin sections and 35-μm thickness for cryosections before being processed for immunofluorescence staining. Primary antibody incubations were conducted overnight at 4 °C with antibodies targeting NLRP3 (1:100; Cell Signaling Technology, D4D8T, MA, USA), caspase-1 (1:100; Adipogen, Casper-2, San Diego, CA, USA), and HMGB1 (1:100; Abcam, ab79823, UK). For immunofluorescence, the sections were then incubated with Cy3-conjugated (1:200; Abcam, ab6939/ab97035, UK) or Alexa Fluor 488-conjugated (1:300; Abcam, ab150077/ab150113, UK) secondary antibodies. Nuclei were stained with 4’,6-diamidino-2-phenylindole (DAPI; Beyotime, China). The slices were then washed and mounted with UltraCruzTM mounting medium (Santa Cruz Biotechnology; Dallas, TX, USA). After incubation with primary and secondary antibodies, images were acquired with a laser scanning confocal fluorescence microscope (Leica TCS SP8 Confocal Laser Scanning Microscope). We measured 3–4 mice from different groups at each time point, with 3 slices per mouse and 1 field per slice. Location of the brain slices which was selected for the analysis of immunofluorescence staining by referring to stereotaxic atlas of the mouse brain. In evaluating the positive cells, a 500 × 500-pixel rectangular ROI (details in Additional file 1: Fig. S2) was selected from each image. Cells were considered positive for NLRP3, pro-caspase 1, and HMGB1 if specific fluorescence was observed. Nuclei were after stained DAPI to show total cells. We used previously described methods [19, 20] and Image-Pro Plus 6.0 software (Media Cybernetics, Rockville, MD, USA) to analyze the results. The “% Cell+ ” was defined as the percentage of NLRP3, pro-caspase 1, and HMGB1 positive cells relative to the total number of brain cells. Z-stack was not acquired.

### Behavioral tests

A T-maze test was conducted following a previous protocol [21] to test the working memory of the mice. A nesting test was performed as previously described [22] to test the social behavior of the mice. Novel object recognition experiments were performed in a home-cage system (Noldus Information Technology) according to previous methods [23, 24] to measure short-term memory changes in mice. The relative exploration time (t) is recorded and expressed as a discrimination index (DI = (*t*_novel_ − *t*_familiar_)/(*t*_novel_ + *t*_familiar_) × 100%).

### Western blot assays

To minimize the influence of anesthesia and hypothermia on NMDAR1 phosphorylation, the mice were rapidly decapitated without anesthesia for western blot analysis. Western blot analysis was conducted using fresh and unfixed contralateral hippocampi and contralateral prefrontal cortex obtained from mice at Western blot analysis of hippocampal and PFC homogenates was also performed weeks and 8 weeks after TBI. Hippocampal and PFC specimens were suspended in 0.4 ml of protease-phosphatase inhibitor buffer and homogenized in an ice-cold environment. The protease-phosphatase inhibitor buffer was a mixture of one tablet of Protease and Phosphatase Inhibitor Mini Tablets (Pierce, Rockford, USA) per 10 ml of T-PERTM Tissue Protein Extraction Reagent (Pierce, Rockford, IL, USA). Each tablet contains a mixture of several potent inhibitors, including aprotinin, bestatin, E-64, leupeptin, sodium fluoride, sodium orthovanadate, sodium pyrophosphate, β-glycerophosphate and EDTA. After normalization, the samples were subjected to polyacrylamide gel electrophoresis (10% gel, Genscrpit, NJ, USA) and transferred onto an Immobilon-P PVDF membrane (GE Healthcare, Chicago, IL, USA). Immunoblot analysis was performed to detect NLRP3 (1:1000; Adipogen, Cryo-2, USA), ASC (1:1000; Adipogen, AL177, USA), pro-caspase-1 (1:1000; Abcam, ab179515, UK), caspase-1 (1:1000; Adipogen, Casper-2, USA), HMGB1 (1:1000; Abcam, ab79823, UK), and GAPDH (Bioworld, USA) and phosphorylation of NMADAR1 at Ser896 (1:500; Abcam, ab68144, UK) as well as total NMDAR1 (1:1000; Cell Signaling Technology, D65B7, USA). After being probed with horseradish peroxidase-conjugated secondary antibody, the membranes were visualized with Super Signal Chemiluminescent Substrates (Bio-Rad, CA, USA). Panels were analyzed using ImageJ (National Institutes of Health, USA, ver 1.8.0).

### Analysis of cytokines via ELISA

IL-1β, IL-18, TNF-α, and HMGB1 concentrations in supernatant samples from cortical homogenates were assessed according to the instructions of ELISA kits (Sino Biological, China). Briefly, duplicate samples or standards (50 μL/well) were added, and plates were incubated (24 h, 4 ºC) and washed before the addition of detection antibody (biotinylated goat anti-mouse antibody) for 1 h at room temperature. The plates were washed, incubated with the streptavidin–horseradish peroxidase conjugate (20 min, room temperature), and washed again before the addition of substrate solution. After color development, the reaction was stopped by the addition of 1 M H_2_SO_4_ (25 μL), and the plates were read at 450 nm (Labsystem Multiskan RC, UK).

### Primary culture of hippocampal cells and recombinant HMGB1 treatment

Mouse hippocampal neuron and microglial cocultures were prepared as previously described [25]. In brief, embryos were obtained from 18-day gestating mice anaesthetized under pentobarbital sodium. The hippocampus was isolated using sterile micro-forceps under a stereomicroscope and treated with 0.25% trypsin for 15 min at 37 °C. Trypsinization was stopped by the addition of 10% FBS, and cell suspensions were seeded at 1 × 10^5^ cells cm^−2^ in neurobasal medium (Invitrogen, USA) containing 2% B27 supplement (Invitrogen, USA), 0.5 mM l-glutamine and 25 μM L-glutamic acid. Four days later, half of the medium was replaced with B27/neurobasal medium without l-glutamic acid. The cells were used after 14 days of culture. Recombinant HMGB1 (Sino Biological, Beijing, China) was added to the medium at a concentration of 0.50 ng/mL or 100 ng/mL for 24 h.

### Glycyrrhizin injection

Before use, glycyrrhizin (Selleck Chemicals LLC, USA) was dissolved in 50 mM NaOH at 37 °C, and the pH was adjusted to 7.4 using 0.5 M Tris–HCl buffer (pH 6.8). Immediately after the wound was sutured, glycyrrhizin (1 mg/kg) was injected intraperitoneally and then administered intraperitoneally daily for one week. The dose was determined as described previously [26].

### Electrophysiological recording of synaptic plasticity in hippocampal slices

The experiments were performed as described previously [27]. Briefly, mice were deeply anaesthetized with urethane (25%, 1 ml/100 g) and decapitated. The electrode impedance after filling the recording electrode fluid was 1 to 2 MΩ. The stimulation electrode impedance was 500 KΩ to 1 MΩ. (GB 150F-8P, Sutter Instrument, USA). The ion currents were recorded with an Axopatch 700B amplifier. After digitization of the amplified and filtered (low-pass filter 1 kHz) signals with a Digidata 1440A transducer, pClamp10.6 was used for acquisition of the recording. The data were analyzed off-line using the same software. The recording electrode was placed at layer IV at 170 to 190 µm, approximately 1/2 of the thickness of the cortex, to record the fEPSP. The data were digitized at 3 kHz and analyzed with Clampfit 10.6 software. Stimulus–response curves were performed at the beginning of each experiment, and pulses at an intensity eliciting 40–50% of a maximal response were delivered as the baseline level. The slices were stimulated with single test pulses every 30 s for at least 20–30 min followed by high-frequency stimulation (HFS) and 60 min of test stimulation.

The time course of changes in the fEPSP amplitudes was calculated in relation to the signals obtained during the last 10 min prior to HFS (100%), and all responses were normalized to this baseline and then averaged across experiments. The degree of long-term potentiation (LTP) is expressed as a percentage of the original control level. All changes in long-term synaptic plasticity were evaluated by averaging 20 responses at 81–90 min post-HFS and comparing these data to the 20 control signals during the last 10 min prior to HFS.

### Statistical analysis

The results are expressed as the mean ± SD. All data passed the Kolmogorov–Smirnov test for normality (*p* > 0.1000). All experimental assessments were conducted by lab technicians who were blinded to the genotype and treatment of the experimental animals. Additionally, all results from this study were analyzed by researchers who were also blinded to the experimental grouping. Differences between two groups were analyzed using Student’s t-test or a rank sum test for discontinuous variables, and statistical comparisons of more than two groups were performed using factorial ANOVA followed by Bonferroni’s post hoc test. Two-way analyses of variance (ANOVAs) were used to assess the effects of genotype (or treatment), day, and the genotype (or treatment) × day interaction. Prism GraphPad 8.0 was used for analysis and graph.

## Results

### The NLRP3 inflammasome is continuously activated in the hippocampus–prefrontal cortex circuit for up to 8 weeks after TBI, followed by elevated levels of HMGB1

**Fig. 1:**
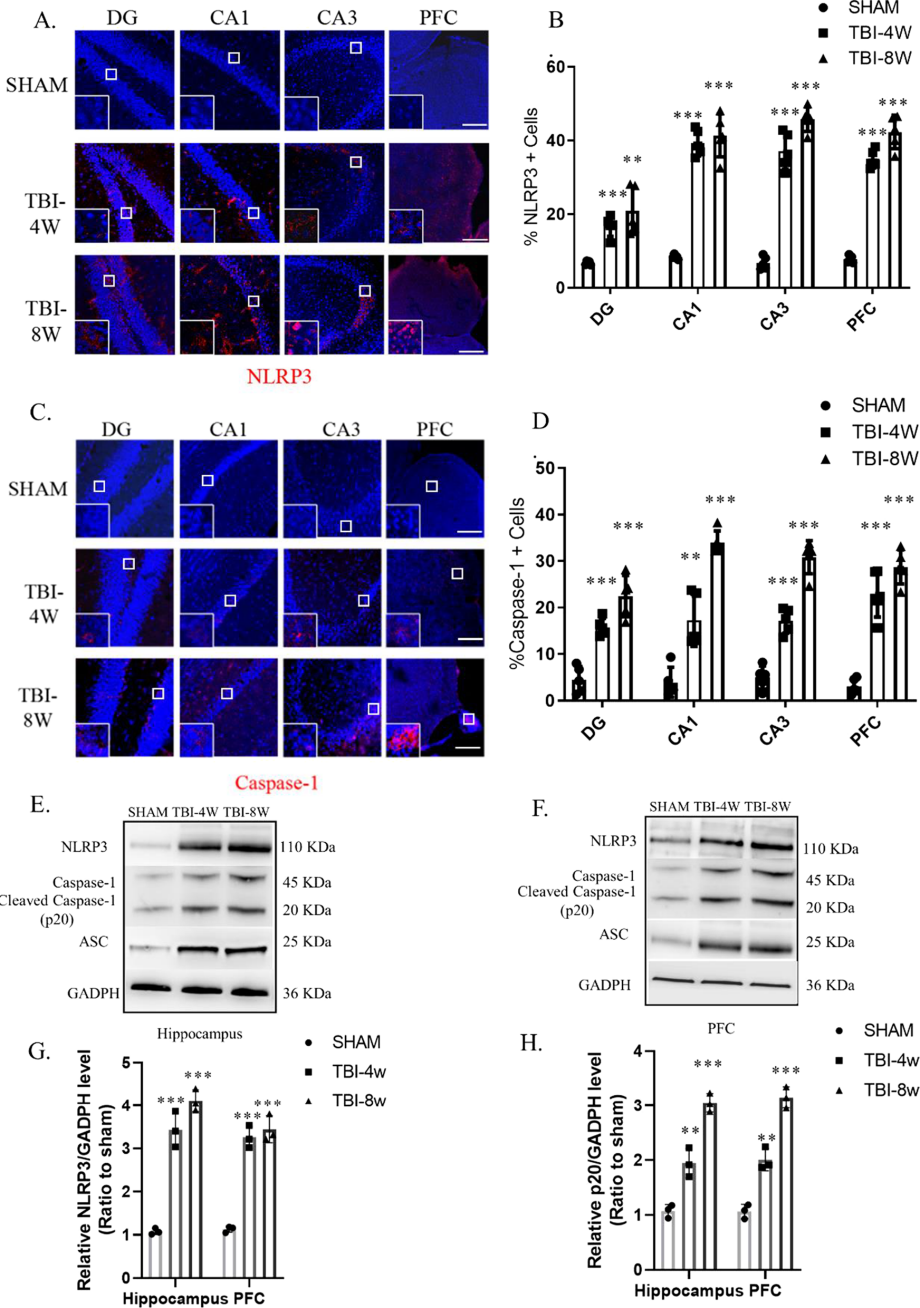
The NLRP3 inflammasome is activated in the hippocampus–prefrontal cortex circuit 4 weeks to 8 weeks post-TBI. **A**–**D** Immunofluorescence revealed that NLRP3 (**A**–**B**) and caspase-1 (**C**–**D**) were significantly activated in the hippocampal DG, CA1, and CA3 and the prefrontal cortex at 4 weeks and 8 weeks post-TBI. Data represent the means ± SD (*n* = 5 mice per group). Representative protein bands showing NLRP3, ASC, pro-caspase-1, and caspase-1 (P20) expression in the hippocampus (**E**) and prefrontal cortex (**F**). **G**–**H** Western blot analysis indicating the level of the NLRP3 inflammasome in the hippocampus and prefrontal cortex. Each experiment was repeated three times. Data were normalized to Sham group data and are presented as the means ± SD (*n* = 3 mice per group). 2-way ANOVA, *p < 0.05, **p < 0.01, ***p < 0.001, compared to the Sham group

Because the injured lateral hippocampus turned into liquefactive necrosis after 4 w, the contralateral hippocampus and PFC were used in this study. After immunofluorescence detection of key elements, namely, NLRP3 and pro-caspase-1, we found that the hippocampus and the PFC at 4 w after TBI (Fig. 1A–D) showed significantly high levels of the NLRP3 inflammasome, which continued for 8 w after injury. Western blot analysis showed that cleaved caspase-1 (p20) protein levels were significantly higher at 8 w than at 4 w (compared to the sham operation group), which suggests continuous NLRP3 inflammasome activation. Immunofluorescence results showed that HMGB1 levels in the hippocampal DG, CA1, and CA3 and the PFC were significantly increased at 4 w after TBI (Fig. 4A–B) and were even higher at 8 w after TBI (Fig. 4B). Western blot analysis of hippocampal and PFC homogenates was also performed (Fig. 4C). Compared to the sham group, HMGB1 protein levels were significantly higher at 4 w after TBI, and significantly higher at 8 w compared to 4 w. Western blot results were consistent with immunofluorescence.

### NLRP3 knockout improves chronic impairment of short-term memory, working memory and social behavior after TBI

**Fig. 2:**
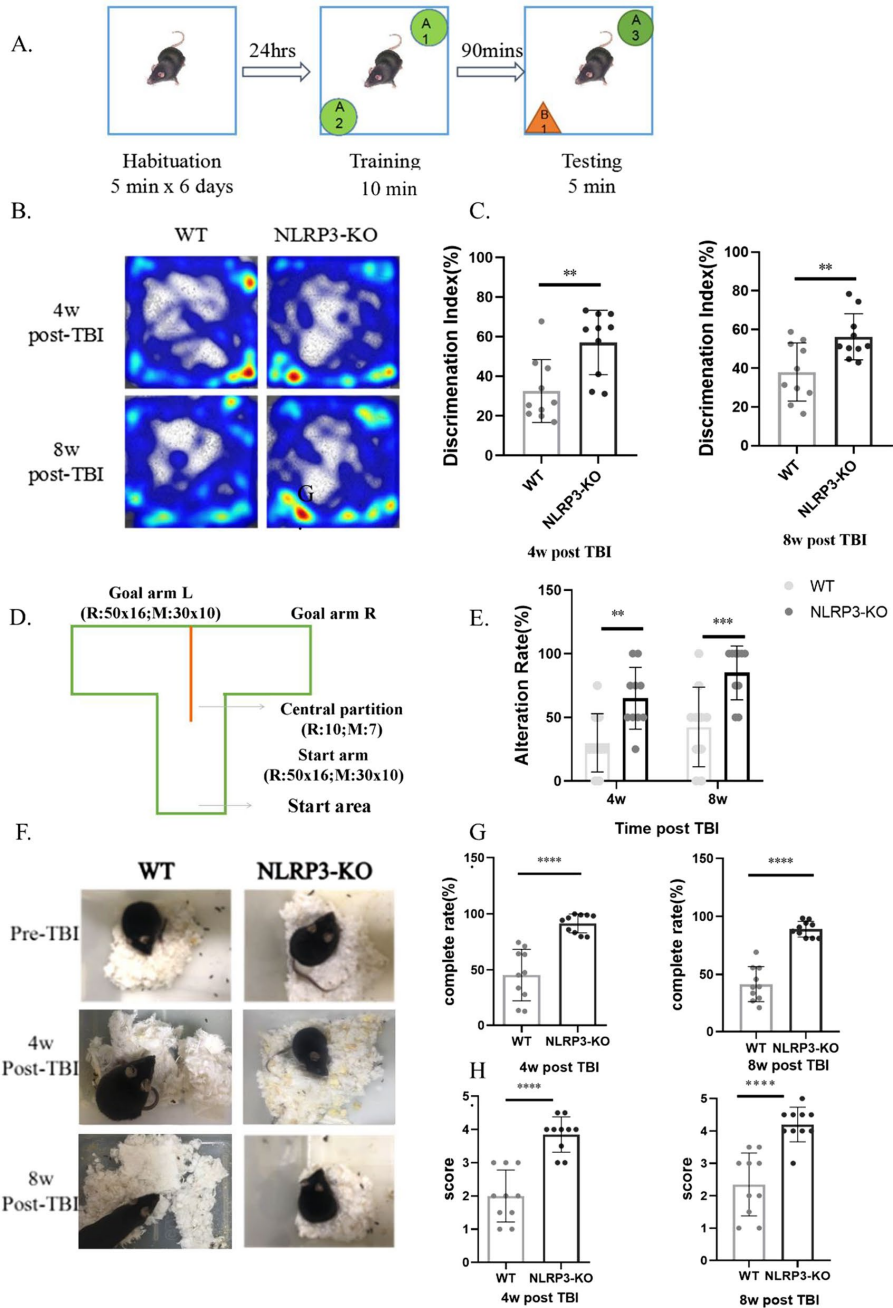
NLRP3 knockout improves cognitive performance in the late stage of TBI. **A** Schematic representation of the method and process for the new object recognition (NOR) test. **B** Representative heatmap results for the NOR test indicating better short-term memory of NLRP3 knockout mice than wild-type mice. **C**–**D** Discrimination index of wild-type and NLRP3 knockout mice in the NOR test at 4 weeks and 8 weeks post-TBI, Unpaired t-test. **D** Schematic representation of the apparatus used in the T-maze test. **E** Alternation rate (%) in the T-maze test indicating that NLRP3 knockout attenuated working memory impairment at 4 weeks and 8 weeks post-TBI,2-way ANOVA. **F** Representative results of the nest-building assay. Complete rate (%) (**G**) and score (**H**) indicating that NLRP3 knockout attenuated social behavior at 4 weeks and 8 weeks post-TBI, unpaired t-test. Data represent the means ± SD (*n* = 10 mice per group). **p < 0.01, ***p < 0.001, ****p < 0.0001 compared to the WT group

To detect behavioral changes in mice 4 w and 8 w after TBI, we used three methods: new object recognition, the T-maze test, and the nest building test. In the new object recognition experiment, there was no significant difference in locomotor activity of the mice during the habituation training phase, whereas evaluated short-term memory (Fig. 2A), and the residence time of WT mice (4 w and 8 w after injury) with new objects was less than that of NLRP3-KO mice (Fig. 2B). The discrimination indexes (Fig. 2C) of WT mice were also significantly lower than those of NLRP3-KO mice, suggesting that NLRP3 knockout significantly alleviated TBI-induced short-term memory impairment. In the T-maze test (Fig. 2D), which was designed according to Deacon et al. [21], the alternation rate of NLRP3-KO mice at 4 w and 8 w after injury (Fig. 2E) was significantly better than that of WT mice. Nest building is a high-level cognitive function that relies on the hippocampus and can reflect changes in working memory and social behavior in rodents [22]. Results of the nesting experiments revealed that the nest shape (Fig. 2F), nest completion degree (Fig. 2G) and score (Fig. 2H) of NLRP3-KO mice were significantly better than those of WT mice. Meanwhile, the sham groups of NLRP3-KO and WT mice did not differ significantly in the above behavioral tests (Additional file 1: Fig. S1 A–G).

### The reduced amplitude of LTP in the PFC and increased HMGB1 levels can be eliminated by NLRP3 knockout

**Fig. 3:**
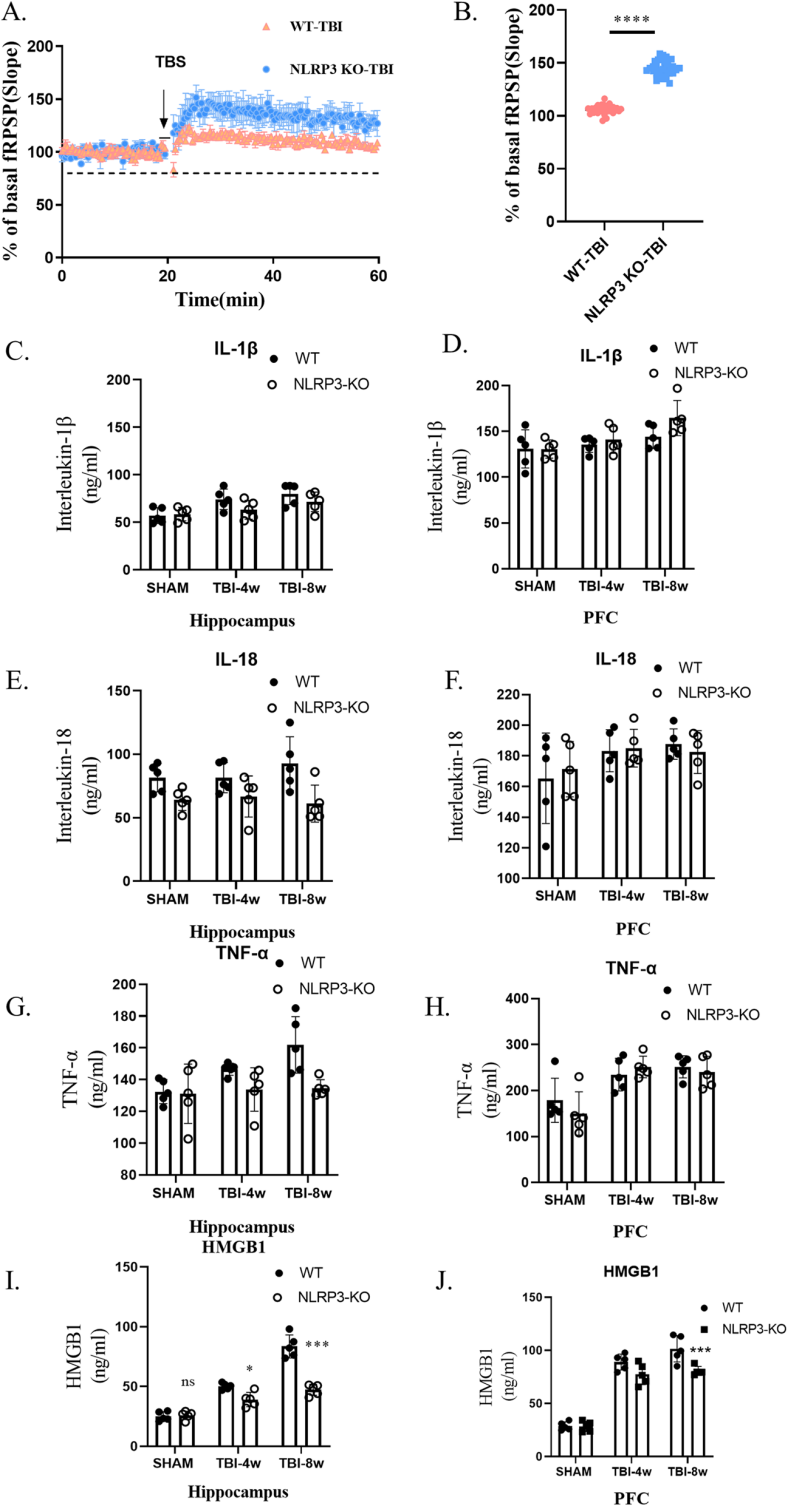
NLRP3 knockout significantly improved LTP amplitude without a significant reduction in IL-1β, IL-18 or TNF-α levels. **A** Slope changes in fEPSP in the prefrontal cortex at 4 weeks post-TBI. **B** NLRP3 knockout significantly improved LTP amplitude in the prefrontal cortex at 4 weeks post-TBI. Data represent the means ± SD (*n* = 9 slopes per group), unpaired t-test. ****p < 0.0001 compared to the WT group. **C**–**H** ELISA results for IL-1β, IL-18 and TNF-α levels in hippocampal and prefrontal cortex tissue, 2-way ANOVA. Data represent the means ± SD (*n* = 3 per group). ns (*p* > 0.05) compared to the WT group. Each experiment was repeated three times

Due to the large necrotic area in the injured hippocampus 4 w after TBI, we only tested the LTP of the PFC. We performed electrophysiological recordings of synaptic plasticity in PFC slices from mice 4 w after TBI and found that the LTP amplitude in NLRP3-KO mice was significantly higher than that in WT mice (Fig. 3A, B), which confirmed that timepoint cognitive impairment does exist and indicated that NLRP3 knockout has a protective effect on TBI-induced cognitive impairment of the hippocampus-PFC circuit.

We measured the levels of NLRP3 inflammasome-related inflammatory factor factors in the hippocampus and PFC using ELISA kits. Compared to WT mice, NLRP3-KO mice showed no differences in the levels of IL-1β (Fig. 3C), IL-18 (Fig. 3D) and TNF-α (Fig. 3E). However, HMGB1 levels were significantly reduced after NLRP3 knockdown (Fig. 3F).

### HMGB1 antagonism improved the long-term potentiation amplitude and cognitive impairment and inhibited NLRP3 inflammasome activation

**Fig. 4:**
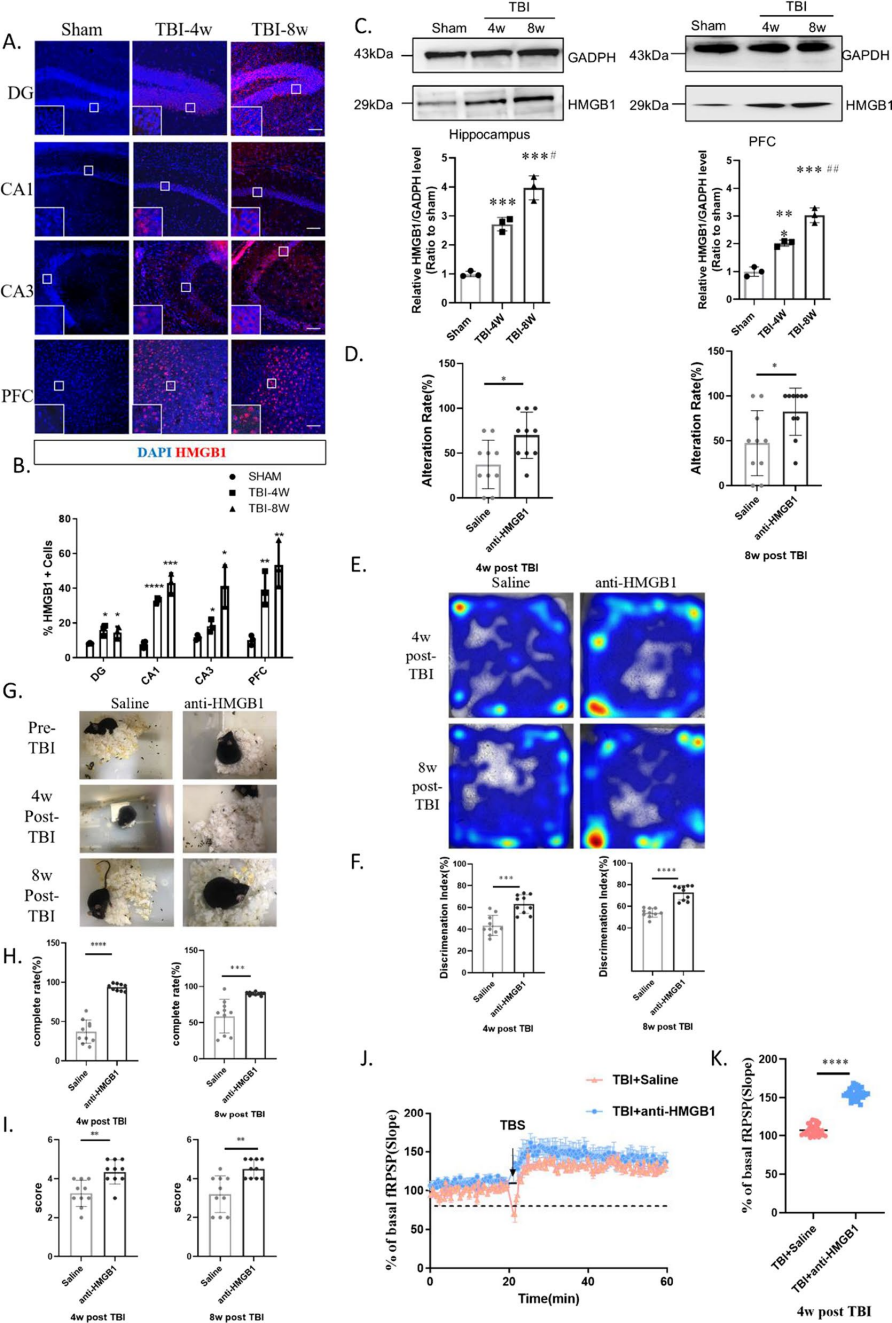
Upregulation of HMGB1 in the hippocampus–prefrontal cortex circuit caused LTP inhibition and cognitive dysfunction. **A**–**B** Immunofluorescence revealed that HMGB1 was significantly upregulated in the hippocampal DG, CA1, and CA3 and the prefrontal cortex at 4 weeks and 8 weeks post-TBI. Data represent the means ± SD (*n* = 3 mice per group). 2-way ANOVA, *p < 0.05, **p < 0.01, ***p < 0.001, ****p < 0.0001 compared to the Sham group. Scale bar: 50 µm. **C** Western blot analysis indicating that the level of HMGB1 was significantly upregulated in the hippocampus–prefrontal cortex circuit 4 weeks and 8 weeks post-TBI. Data represent the means ± SD (*n* = 3 per group). 2-way ANOVA,***p < 0.001 vs. Sham; #*p* < 0.05, ##*p* < 0.01 vs. TBI-4w. Each experiment was repeated three times. (D)Glycyrrhizin treatment significantly improved working memory assessed by T-maze at 4 weeks and 8 weeks post-TBI, 2-way ANOVA. **E**–**F** Representative hot zone results and discrimination index of two groups in the NOR test, Unpaired t-test. **G** Representative results of the nest-building assay. Complete rate (%) (**H**) and score (**I**) indicating that glycyrrhizin treatment attenuated social behavior. ****p < 0.0001 at 4 weeks and 8 weeks post-TBI. Data represent the means ± SD (*n* = 10 mice per group). Unpaired t-test, **p < 0.01, ***p < 0.001, ****p < 0.0001 compared to the WT group. **J**–**K** Glycyrrhizin treatment significantly improved LTP amplitude in the prefrontal cortex at 4 weeks post-TBI, Unpaired t-test. Data represent the means ± SD (*n* = 9 slopes per group compared to the WT group

**Fig. 5:**
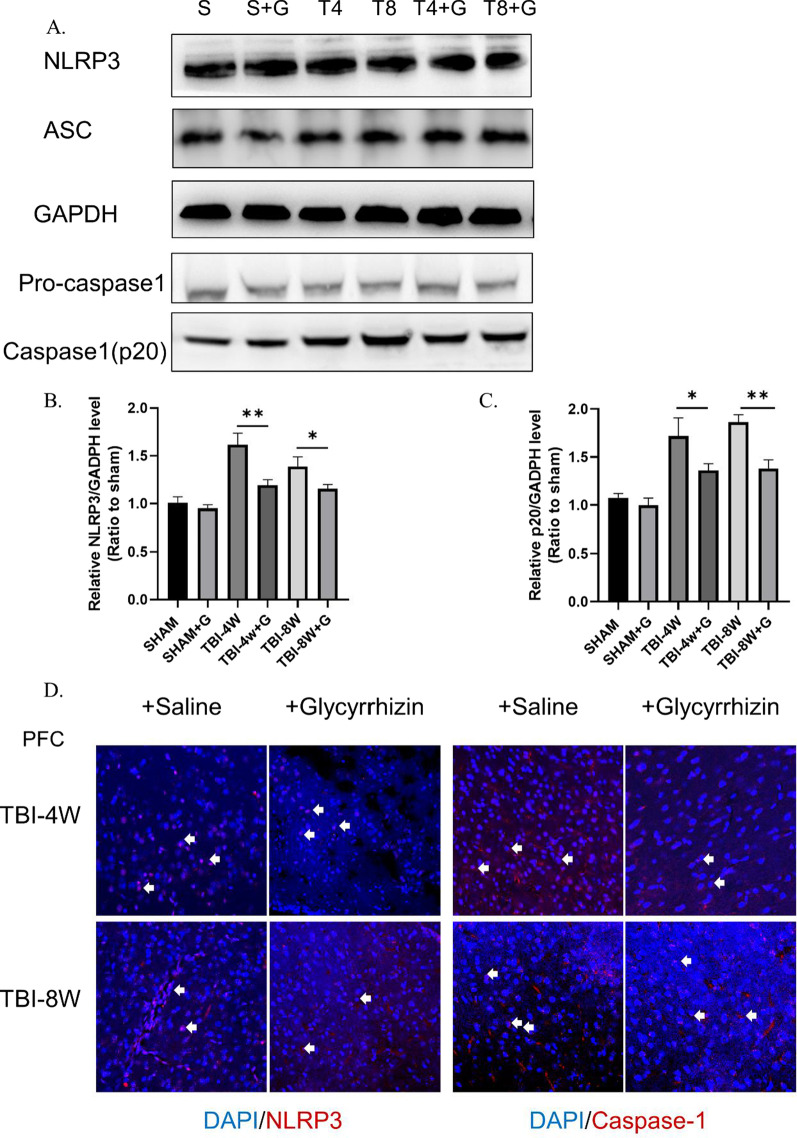
Glycyrrhizin inhibits the activation of NLRP3 inflammasome. **A**–**C** Western blot band and analysis indicating that the level of NLRP3 and caspase-1 (p20) were significantly reduced in TBI groups. Data represent the means ± SD (*n* = 3 per group), Unpaired t-test. *p < 0.05, **p < 0.01 compared to the sham group (**D**). Immunofluorescence revealed that NLRP3 and Caspase-1 level were significantly reduced by glycyrrhizin treatment

To verify whether HMGB1 is a key molecule in impaired cognitive behavior, we administered glycyrrhizin to TBI model mice via intraperitoneal injection for 7 consecutive days beginning immediately after injury (see Additional file 1: Fig. S1 H for the timeline of drug administration and testing). The working memory performance of treated mice in the T-maze test (Fig. 4D) was significantly better than that of vehicle control group mice. In the new object recognition test (Fig. 4E), the Discrimination Index (DI%) of the glycyrrhizin-treatment group was significantly better than that of the saline group (Fig. 4F). The completeness of nest building and the scores of the mice at 4 w and 8 w (Fig. 4G–I) after injury were significantly better than those of mice in the saline group. LTP analysis showed a nearly 50% (mean, 106.92 vs. 154.85) improvement in the PFC (Fig. 4J–K). The results of immunofluorescence (Fig. 5A–B) and immunoblotting (Fig. 5C–D) assays showed that NLRP3, ASC, and caspase-1 (p20) levels were significantly decreased after antagonizing HMGB1 with glycyrrhizin.

### Recombination of HMGB1 reduced p-NR1 (Ser896), while both NLRP3 KO and HMGB1 antagonism improved p-NR1 levels after TBI

**Fig. 6:**
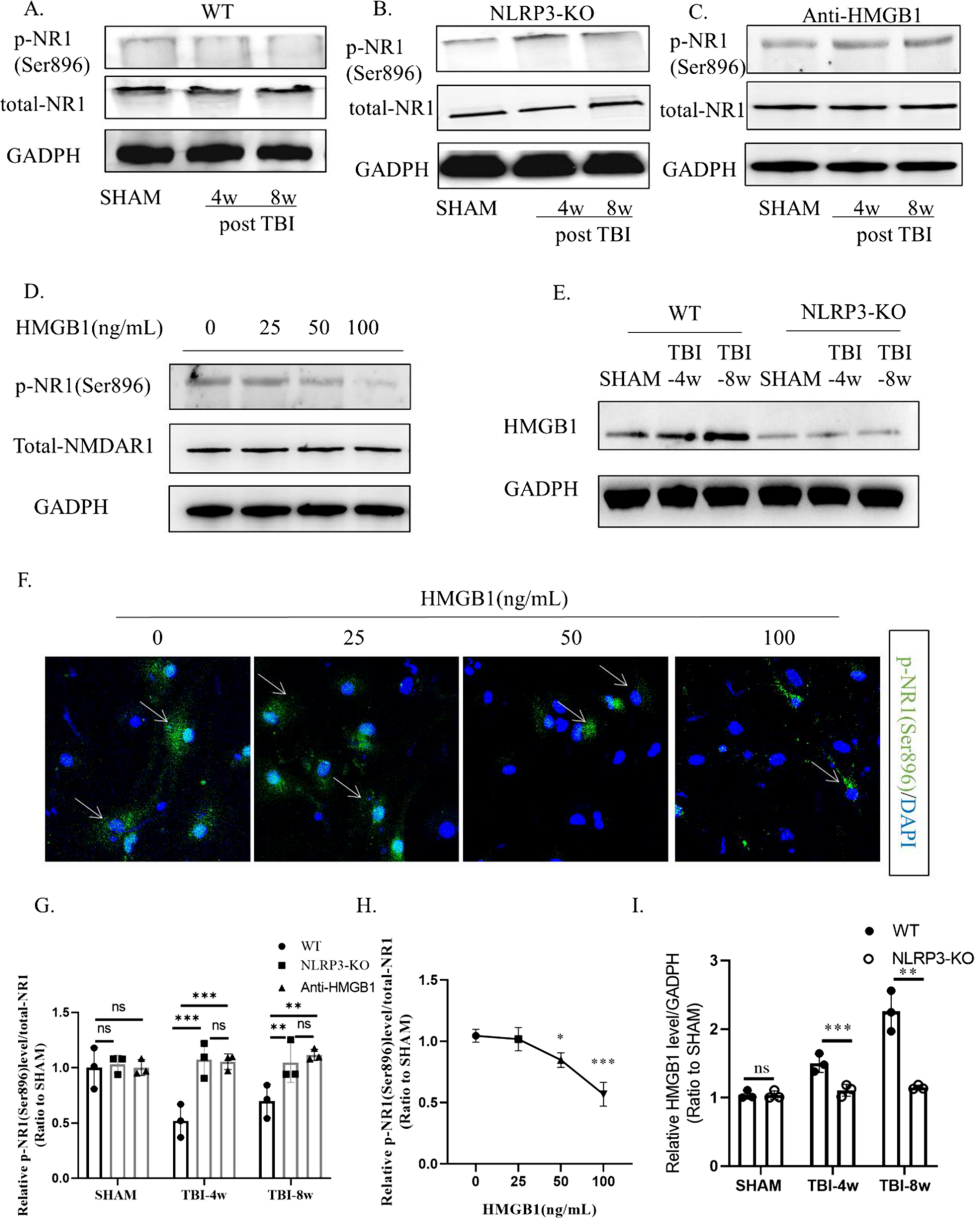
HMGB1 antagonism reduced p-NR1 levels after TBI. Representative protein bands showing HMGB1 expression in wild type (**A**), NLRP3 knockout mice (**B**), and the glycyrrhizin treatment group (**C**) 4 weeks and 8 weeks post-TBI. Western blot analysis (**F**) indicating that NLRP3 knockout significantly rescued the decline in p-NR1 (Ser896) levels. 2-way ANOVA. **C**, **G** The phosphorylation level of NR1 at Ser896 was regulated by an HMGB1 concentration gradient in mixed neuronal cell culture (**D**, **H**). Scale bar: 50 μm. NLRP3 knockout significantly reduced HMGB1 secretion (**E**, **I**). Data represent the means ± SD (*n* = 3 per group). 2-way ANOVA, ns (*p* > 0.05), **p < 0.01, ***p < 0.001 compared to the WT group or no-HMGB1 group. Each experiment was repeated three times

The phosphorylated NMDAR1 (p-NR1) level in WT mice continued to decrease at 4 w and 8 w after TBI (Fig. 6A, I); NLRP3 knockout improved this downward trend (Fig. 6B, I), and glycyrrhizin treatment effectively alleviated the decrease in p-NR1 levels (Fig. 6C, G). Moreover, unlike canonical inflammatory factors, such as IL-1β, HMGB1 levels were significantly lower in NLRP3-KO mice than in WT mice (Fig. 6E, I). We then used recombinant (mouse derived) HMGB1 as a cytokine to stimulate hippocampal neurons cultured in vitro. Western blot analysis of cell homogenates revealed that when the concentration of HMGB1 reached 50 ng/mL and 100 ng/mL, the level of p-NR1 (Ser896) was significantly reduced (Fig. 6D, H). Immunofluorescence detection showed that the p-NR1 level decreased with increasing HMGB1 concentrations (Fig. 6F).

## Discussion

### The NLRP3 inflammasome and HMGB1 increased in the late stage of TBI

Our data showed that the NLRP3 inflammasome continues to be activated along with high HMGB1 levels in the model at 4–8 weeks after TBI, which is consistent with cognitive impairment and LTP inhibition. The NLRP3 inflammasome and HMGB1 are especially upregulated in the hippocampus–PFC circuit, which is a key brain area for the formation of short-term, working, and social memory. The vulnerable brain areas we found here are consistent with previous studies of an acute TBI model (< 7 days) [28, 29]. In addition, our results are supported by a recent report on NLRP3 inflammasome activation at 3 months post-TBI [30]. Previous case reports have shown that HMGB1 continues to be highly expressed in the injured brain tissue of patients for up to 20 days after injury. In the present study, a high HMGB1 level was observed until 8 weeks post-TBI, while the level of IL-1β was not elevated. However, HMGB1 levels were significantly decreased after NLRP3 knockdown, suggesting that NLRP3 inflammasome activation is closely related to HMGB1 levels in the late stage of TBI.

### Both NLRP3 KO and HMGB1 antagonism ameliorated post-injury cognitive function, probably by improving NR1 phosphorylation

Our study showed that NLRP3-KO mice in the late stage of TBI had significantly larger LTP amplitudes than WT mice, which was consistent with their behavioral performance. In an obese mouse model, NLRP3 knockout significantly improved the LTP amplitude and behavior in the Y-maze [31]. Several researchers also used the same NLRP3-knockout strain as ours, which indicates that deficiency of NLRP3 also alleviates cognitive function impairments in Alzheimer's disease [32], Parkinson's disease [33] and 24 h post-TBI [34]. Consistent with our results, the deficiency of NLRP3 significantly improved the performance of mice in novel object recognition experiments 24 h after TBI, along with decreased inflammatory infiltrate and edema compared to TBI-WT mice. Taken together, these findings suggest that NLRP3 may influence cognitive function and behavioral testing performance by modulating synaptic plasticity.

Although we must note that glycyrrhizin has broad anti-inflammatory properties against not only HMGB1 but also IL-1β, the level of IL-1β was very low compared to that of HMGB1 in our TBI model during the testing period. Thus, like many other investigators, we preferred to consider glycyrrhizin as a relatively specific HMGB1 antagonist here. Consistent with our findings, several studies have found that the use of glycyrrhizin to inhibit HMGB1 can significantly improve cognitive function [14, 35]. Our study found that can significantly improve post-injury LTP, providing evidence that high HMGB1 levels may impair synaptic plasticity during the late stage of TBI.

Many factors affect LTP amplitude, among which anchoring and internalization of NMDARs in the postsynaptic membrane is one of the basic factors [36]. The NR1 subunit is an essential subunit of NMDAR. The phosphorylation level of Ser896 determines the amount of NMDAR1 anchored in the postsynaptic membrane and affects the LTP amplitude [37, 38]. Therefore, inhibition of p-NR1 (Ser896) levels by HMGB1 may mediate NLRP3 inflammasome-induced cognitive impairment. HMGB1 indirectly impairs LTP and memory function in mice in a TLR4- and RAGE-dependent manner and is associated with the excessive activation of JNK and NF-kB [11, 39]. Here, our in vitro experiments confirmed that HMGB1 may inhibit p-NR1 to affect synaptic plasticity more directly. The maturation and active release processes of HMGB1 are closely related to the NLRP3 inflammasome [10]. In our study, knocking out NLRP3, similar to the results in the glycyrrhizin-treatment group, improved LTP and TBI-induced cognitive impairment. Therefore, HMGB1 may be an important reason for NLRP3 inflammasome-induced memory impairment in the late stage of TBI.

### HMGB1 may be an effective target for the treatment of cognitive impairment after TBI

As an important member of DAMP, HMGB1 has different functions in different subcellular localization. In TBI, HMGB1 can be largely released from necrotic neurons [10]. Meanwhile, HMGB1 can also be secreted extracellularly to mediate the amplification of neuroinflammation. In this study, we found that HMGB1 was predominantly localized in the nucleus at 4 weeks post-injury, whereas there was significant cytoplasmic and extracellular localization at 8 weeks. This translocation indicated the inflammatory mediator function activity of HMGB1, and by inhibiting HMGB1, the cognitive impairment effect of the NLRP3 inflammasome was rescued, which indicated that in addition to knocking out NLRP3 inflammasomes, targeting HMGB1 can be effective for cognitive protection.

**Fig. 7:**
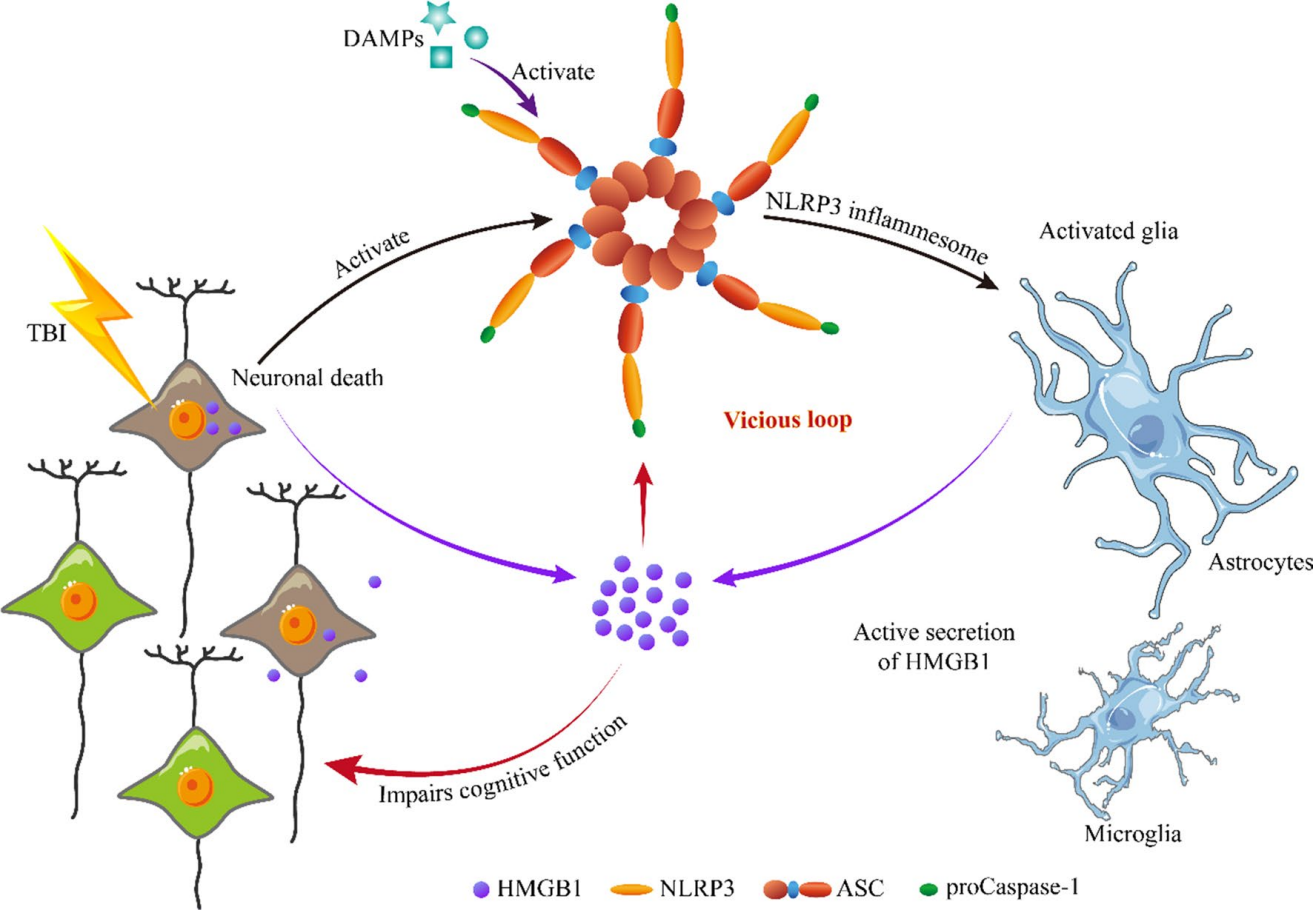
Schematic diagram illustrating the self-reinforcing vicious loop between the NLRP3 inflammasome and HMGB1 occurs in the late stage of TBI

More importantly, we proved that antagonizing HMGB1 resulted in the inhibition of NLRP3 inflammasome activation. This suggests that a self-reinforcing vicious loop between the NLRP3 inflammasome and HMGB1 occurs in the late stage of TBI (schematically illustrated in Fig. 7), which could be an essential but subtle cause of long-term progressive cognitive impairment induced by TBI. This finding is helpful also to explain why chronic traumatic encephalopathy caused by repeated mild TBIs is so difficult to treat.

However, glycyrrhizin is a relatively broad-spectrum anti-inflammatory agent. Thus, an RNA interference or a rescue experiment (add glycyrrhizin first, then HMGB1 to see if the effect of glycyrrhizin is counteracted) in vitro could further validate our results. We will include this part into the design of future experiments.

Currently, there are no clinical studies on the application of glycyrrhizin to improve cognitive function. A study on depression therapy that used a dose of 150 mg/kg in combination with Escitalopram oxalate tablet to significantly improve clinical symptoms of depression and serum TNF-α levels [40]. The most important side effects of glycyrrhizin are hypertension and hypokalemic-induced secondary disorders. The doses used in our animal experiments were one-third of depression therapy study, which we believe has the potential for safe clinical application. In conclusion, inhibiting HMGB1 that stops self-reinforcing vicious loop from the NLRP3 inflammasome is a perspective treatment strategy of cognitive impairment after TBI.

## Conclusion

Our results show that NLRP3 inflammasome activation is an important cause of cognitive impairment after TBI. More importantly, HMGB1 mediates the NLRP3 inflammasome, which more directly affects the synaptic plasticity of neurons, and it could act as a DAMP to cause sustained activation of the NLRP3 inflammasome. Inhibition of HMGB1 interrupts this vicious loop and will be an effective treatment for cognitive impairment after TBI.

## Supplementary Information

Below is the link to the electronic supplementary material.

**Additonal file 1:** Additional data and tables. **Fig. S1.** Additional data from behavioral experiments. (A-D) Nest building results for wild-type and NLPR3 knockout mice 4 weeks or 8 weeks post-TBI. (E–F) New object recognition results for wild-type and NLPR3 knockout mice 4 weeks or 8 weeks post-TBI. (G) T-maze results for wild-type and NLPR3 knockout mice 4 weeks or 8 weeks post-TBI. (H) The glycyrrhizin administration schedule and route for behavioral tests and the electrophysiological study (EPS). Data represent the means ± SD (*n* = 10 mice per group). ns (*p* > 0.05) compared to the WT group. **Fig. S2.** Location of slices and the brain areas (ROI) selected for the analysis of immunofluorescent in the stereotaxic atlas of the mouse brain. DG (Red), CA1 (Yellow), CA3 (Purple) and PFC (Blue). **Table S1.** Total number of mice used in the experiment. **Table S2.** Sample size calculation.

## Data Availability

The data used to support the findings of this study are available from the corresponding author upon request.

## Abbreviations

ASC: Apoptosis-associated speck-like protein
Casp1: Caspase-1
DAMP: Damage-associated molecular pattern
fEPSP: Field excitatory postsynaptic potential
HFS: High-frequency stimulation
HMGB1: High-mobility group box 1
IL-18: Interleukin-18
IL-1β: Interleukin-1β
JNK: C-Jun N-terminal kinases
KO: Knockout
LTP: Long-term potentiation
NLRP3: NLR family pyrin domain containing 3
NMDAR: N-Methyl-D-aspartic acid receptor
PFC: Prefrontal cortex
p-NR1: Phosphorylated NMDAR1
RAGE: Receptor for advanced glycation end-products
TBI: Traumatic brain injury
TLR4: Toll-like receptor 4
TNF-α: Tumor necrosis factor-α
WT: Wild type

## References

[1] Kobeissy FH, editor. Brain neurotrauma: molecular, neuropsychological, and rehabilitation aspects. Boca Raton (FL): CRC Press/Taylor & Francis; 2015 (cited 2018 Sep 21). http://www.ncbi.nlm.nih.gov/books/NBK299186/26269865

[2] Arciniegas DB, Held K, Wagner P (2002). Cognitive impairment following traumatic brain injury. Curr Treat Options Neurol.

[3] Kraus MF, Susmaras T, Caughlin BP, Walker CJ, Sweeney JA, Little DM (2007). White matter integrity and cognition in chronic traumatic brain injury: a diffusion tensor imaging study. Brain.

[4] Ramlackhansingh AF, Brooks DJ, Greenwood RJ, Bose SK, Turkheimer FE, Kinnunen KM (2011). Inflammation after trauma: microglial activation and traumatic brain injury. Ann Neurol.

[5] Heneka MT, McManus RM, Latz E (2018). Inflammasome signalling in brain function and neurodegenerative disease. Nat Rev Neurosci.

[6] Heneka MT, Kummer MP, Stutz A, Delekate A, Schwartz S, Vieira-Saecker A (2013). NLRP3 is activated in Alzheimer’s disease and contributes to pathology in APP/PS1 mice. Nature.

[7] Wallisch JS, Simon DW, Bayır H, Bell MJ, Kochanek PM, Clark RSB (2017). Cerebrospinal fluid NLRP3 is increased after severe traumatic brain injury in infants and children. Neurocrit Care.

[8] O’Brien WT, Pham L, Symons GF, Monif M, Shultz SR, McDonald SJ (2020). The NLRP3 inflammasome in traumatic brain injury: potential as a biomarker and therapeutic target. J Neuroinflammation.

[9] Shiozaki T, Hayakata T, Tasaki O, Hosotubo H, Fuijita K, Mouri T (2005). Cerebrospinal fluid concentrations of anti-inflammatory mediators in early-phase severe traumatic brain injury. Shock.

[10] Frank MG, Weber MD, Watkins LR, Maier SF (2015). Stress sounds the alarmin: the role of the danger-associated molecular pattern HMGB1 in stress-induced neuroinflammatory priming. Brain Behav Immun.

[11] Mazarati A, Maroso M, Iori V, Vezzani A, Carli M (2011). High-mobility group box-1 impairs memory in mice through both toll-like receptor 4 and Receptor for Advanced Glycation End Products. Exp Neurol.

[12] Gao H-M, Zhou H, Zhang F, Wilson BC, Kam W, Hong J-S (2011). HMGB1 acts on microglia Mac1 to mediate chronic neuroinflammation that drives progressive neurodegeneration. J Neurosci.

[13] Paudel YN, Shaikh MF, Chakraborti A, Kumari Y, Aledo-Serrano Á, Aleksovska K (2018). HMGB1: a common biomarker and potential target for TBI, neuroinflammation, epilepsy, and cognitive dysfunction. Front Neurosci.

[14] Webster KM, Shultz SR, Ozturk E, Dill LK, Sun M, Casillas-Espinosa P (2019). Targeting high-mobility group box protein 1 (HMGB1) in pediatric traumatic brain injury: chronic neuroinflammatory, behavioral, and epileptogenic consequences. Exp Neurol.

[15] Kanneganti T-D, Özören N, Body-Malapel M, Amer A, Park J-H, Franchi L (2006). Bacterial RNA and small antiviral compounds activate caspase-1 through cryopyrin/Nalp3. Nature.

[16] Dai S-S, Zhou Y-G, Li W, An J-H, Li P, Yang N (2010). Local glutamate level dictates adenosine A2A receptor regulation of neuroinflammation and traumatic brain injury. J Neurosci.

[17] Zhao Z-A, Zhao Y, Ning Y-L, Yang N, Peng Y, Li P (2017). Adenosine A2A receptor inactivation alleviates early-onset cognitive dysfunction after traumatic brain injury involving an inhibition of tau hyperphosphorylation. Transl Psychiatry.

[18] Okiyama K, Smith DH, Gennarelli TA, Simon RP, Leach M, McIntosh TK (1995). The sodium channel blocker and glutamate release inhibitor BW1003C87 and magnesium attenuate regional cerebral edema following experimental brain injury in the rat. J Neurochem.

[19] Li W, Dai S, An J, Li P, Chen X, Xiong R (2008). Chronic but not acute treatment with caffeine attenuates traumatic brain injury in the mouse cortical impact model. Neuroscience.

[20] Dai S-S, Wang H, Yang N, An J-H, Li W, Ning Y-L (2013). Plasma glutamate–modulated interaction of A2AR and mGluR5 on BMDCs aggravates traumatic brain injury–induced acute lung injury. J Exp Med.

[21] Deacon RMJ, Rawlins JNP (2006). T-maze alternation in the rodent. Nat Protoc.

[22] Deacon RMJ (2006). Assessing nest building in mice. Nat Protoc.

[23] Vogel-Ciernia A, Matheos DP, Barrett RM, Kramár E, Azzawi S, Chen Y (2013). The neuron-specific chromatin regulatory subunit BAF53b is necessary for synaptic plasticity and memory. Nat Neurosci.

[24] Barrett RM, Malvaez M, Kramar E, Matheos DP, Arrizon A, Cabrera SM (2011). Hippocampal focal knockout of CBP affects specific histone modifications, long-term potentiation, and long-term memory. Neuropsychopharmacol.

[25] Brewer GJ, Torricelli JR, Evege EK, Price PJ (1993). Optimized survival of hippocampal neurons in B27-supplemented Neurobasal, a new serum-free medium combination. J Neurosci Res.

[26] Sun Y, Chen H, Dai J, Wan Z, Xiong P, Xu Y (2018). Glycyrrhizin protects mice against experimental autoimmune encephalomyelitis by inhibiting high-mobility group box 1 (HMGB1) expression and neuronal HMGB1 release. Front Immunol.

[27] Costenla AR, Diógenes MJ, Canas PM, Rodrigues RJ, Nogueira C, Maroco J (2011). Enhanced role of adenosine A2A receptors in the modulation of LTP in the rat hippocampus upon ageing: ageing, adenosine and LTP. Eur J Neurosci.

[28] Xu X, Yin D, Ren H, Gao W, Li F, Sun D (2018). Selective NLRP3 inflammasome inhibitor reduces neuroinflammation and improves long-term neurological outcomes in a murine model of traumatic brain injury. Neurobiol Dis.

[29] Yi HJ, Lee JE, Lee DH, Kim YI, Cho CB, Kim IS, et al. The role of NLRP3 in traumatic brain injury and its regulation by pioglitazone. J Neurosurg. 2019;1–9. 10.3171/2019.6.JNS1954 31561220

[30] Henry RJ, Ritzel RM, Barrett JP, Doran SJ, Jiao Y, Leach JB (2020). Microglial depletion with CSF1R inhibitor during chronic phase of experimental traumatic brain injury reduces neurodegeneration and neurological deficits. J Neurosci.

[31] Guo D-H, Yamamoto M, Hernandez CM, Khodadadi H, Baban B, Stranahan AM (2020). Visceral adipose NLRP3 impairs cognition in obesity via IL1R1 on Cx3cr1+ cells. J Clin Invest.

[32] Ising C, Venegas C, Zhang S, Scheiblich H, Schmidt SV, Vieira-Saecker A (2019). NLRP3 inflammasome activation drives tau pathology. Nature..

[33] Zhang X, Zhang Y, Li R, Zhu L, Fu B, Yan T (2020). Salidroside ameliorates Parkinson’s disease by inhibiting NLRP3-dependent pyroptosis. Aging (Albany NY).

[34] Irrera N, Pizzino G, Calò M, Pallio G, Mannino F, Famà F (2017). Lack of the Nlrp3 inflammasome improves mice recovery following traumatic brain injury. Front Pharmacol.

[35] Yu M, Huang H, Dong S, Sha H, Wei W, Liu C (2019). High mobility group box-1 mediates hippocampal inflammation and contributes to cognitive deficits in high-fat high-fructose diet-induced obese rats. Brain Behav Immun.

[36] Ferreira SG, Gonçalves FQ, Marques JM, Tomé ÂR, Rodrigues RJ, Nunes-Correia I (2015). Presynaptic adenosine A2A receptors dampen cannabinoid CB1 receptor-mediated inhibition of corticostriatal glutamatergic transmission. Br J Pharmacol.

[37] Hida H, Mouri A, Mori K, Matsumoto Y, Seki T, Taniguchi M (2015). Blonanserin ameliorates phencyclidine-induced visual-recognition memory deficits: the complex mechanism of blonanserin action involving D_3_-5-HT_2_A and D_1_-NMDA receptors in the mPFC. Neuropsychopharmacology.

[38] Xu H, Bae M, Tovar-y-Romo LB, Patel N, Bandaru VVR, Pomerantz D (2011). The human immunodeficiency virus coat protein gp120 promotes forward trafficking and surface clustering of NMDA receptors in membrane microdomains. J Neurosci.

[39] Costello DA, Watson MB, Cowley TR, Murphy N, Murphy Royal C, Garlanda C (2011). Interleukin-1alpha and HMGB1 mediate hippocampal dysfunction in SIGIRR-deficient mice. J Neurosci.

[40] Cao Z-Y, Liu Y-Z, Li J-M, Ruan Y-M, Yan W-J, Zhong S-Y (2020). Glycyrrhizic acid as an adjunctive treatment for depression through anti-inflammation: a randomized placebo-controlled clinical trial. J Affect Disord.

