# Supplementary material for: HMGB1 mediates cognitive impairment caused by the NLRP3 inflammasome in the late stage of traumatic brain injury
Source: J Neuroinflammation. 2021 Oct 19;18:241. doi: 10.1186/s12974-021-02274-0 (PMC8527642; doi:10.1186/s12974-021-02274-0)
Supplement: Supplementary file 1 — Additonal file 1: Additional data and tables. Fig. S1. Additional data from behavioral experiments. (A-D) Nest building results for wild-type and NLPR3 knockout mice 4 weeks or 8 weeks post-TBI. (E–F) New object recognition results for wild-type and NLPR3 knockout mice 4 weeks or 8 weeks post-TBI. (G) T-maze results for wild-type and NLPR3 knockout mice 4 weeks or 8 weeks post-TBI. (H) The glycyrrhizin administration schedule and route for behavioral tests and the electrophysiological study (EPS). Data represent the means ± SD (n = 10 mice per group). ns (p > 0.05) compared to the WT group. Fig. S2. Location of slices and the brain areas (ROI) selected for the analysis of immunofluorescent in the stereotaxic atlas of the mouse brain. DG (Red), CA1 (Yellow), CA3 (Purple) and PFC (Blue). Table S1. Total number of mice used in the experiment. Table S2. Sample size calculation. [file 12974_2021_2274_MOESM1_ESM.docx]

**
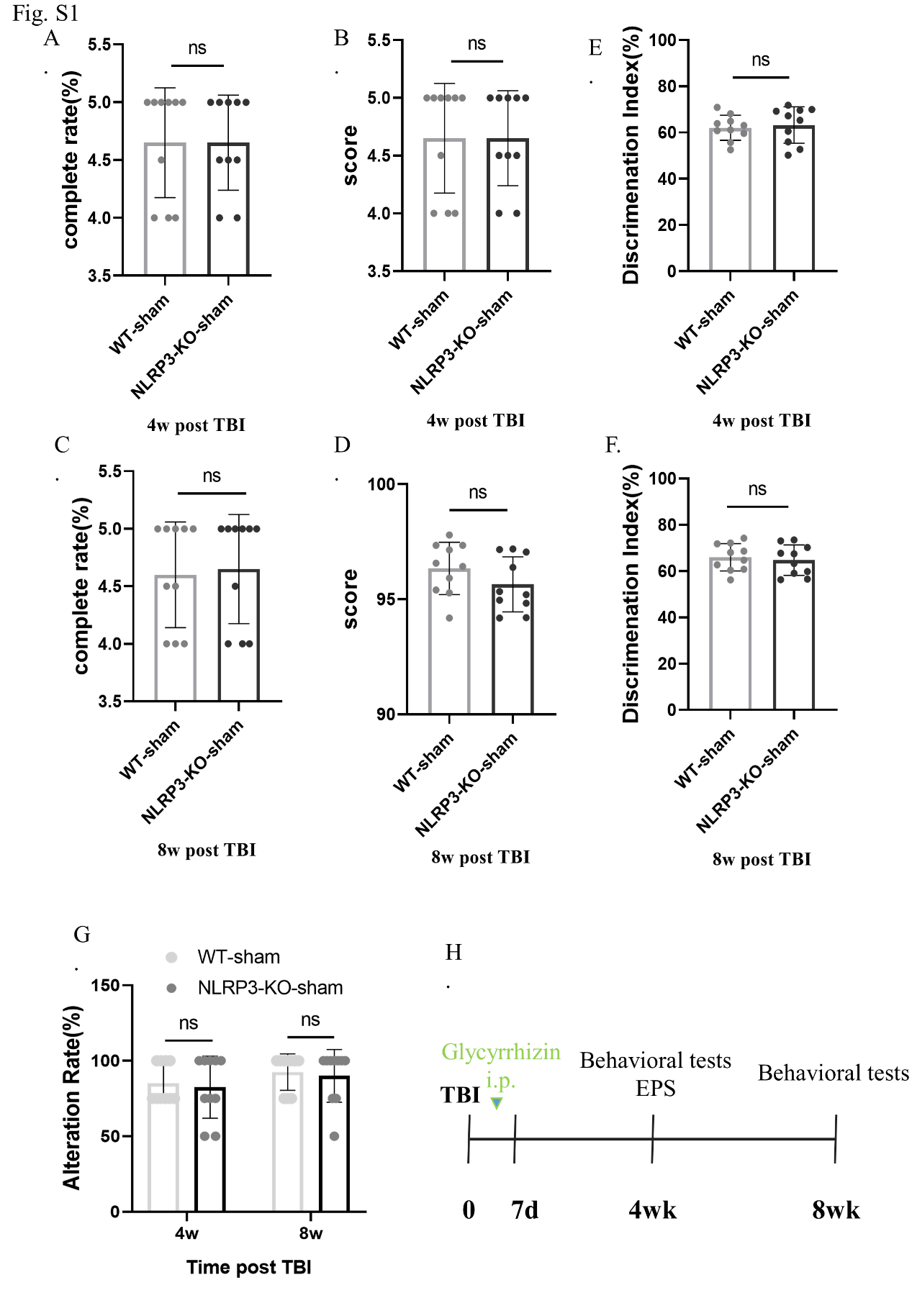
**

**figure S1:** (A-D) Nest building results for wild-type and NLPR3 knockout mice 4 weeks or 8 weeks post TBI. (E-F) New object recognition results for wild-type and NLPR3 knockout mice 4 weeks or 8 weeks post TBI. (G) T-maze results for wild-type and NLPR3 knockout mice 4 weeks or 8 weeks post TBI. (H) The glycyrrhizin administration schedule and route for behavioral tests and the electrophysiological study (EPS). Data represent the means ± SD (n=10 mice per group). ns (p>0.05) compared to the WT group.


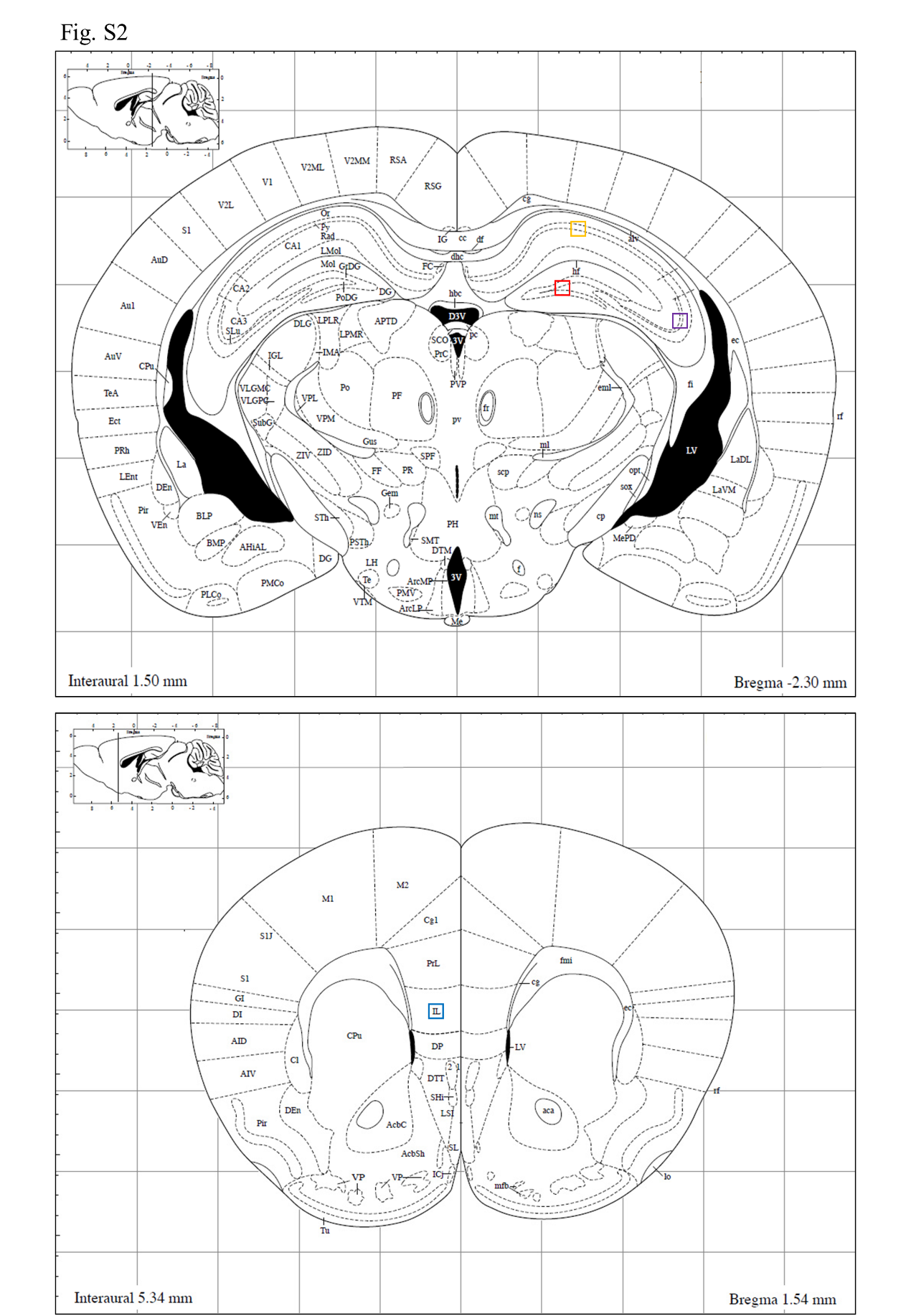


**figure S2:** Location of slices and the brain areas (ROI) selected for the analysis of immunofluorescent in the stereotaxic atlas of the mouse brain. DG (Red), CA1 (Yellow), CA3 (Purple) and PFC (Blue).

**Table 1.**

| Experiments | WT | NLRP3-KO | WT  +saline | WT  +GZ | Pregnant mice |
| --- | --- | --- | --- | --- | --- |
| IF | 18 | 18 | 12 | 12 | 0 |
| WB | 12 | 12 | 15 | 15 | 0 |
| Behavioral test | 30 | 36 | 30 | 33 | 0 |
| ELISA | 12 | 12 | 0 | 0 | 0 |
| Electrophysiological recording | 6 | 6 | 6 | 6 | 0 |
| Primary culture | 0 | 0 | 0 | 0 | 6 |
| Total | 78 | 84 | 57 | 60 | 6 |

**Table S1:** **Total number of mice used in the experiment.** Based on our previous studies and pilot experiments, the mortality, and dropouts of the moderate TBI model was zero.

**Table 2.**

| Pilot Experiments | Mean 1 | Mean 2 | Std 1 | Std 2 | | Cohen's d n  (Effect size) | | |
| --- | --- | --- | --- | --- | --- | --- | --- | --- |
| T-Maze | 75 | 45 | 22.34 | 15.23 | | 1.56 9 | | |
| Nest-building | 81.86 | 53.44 | 26.38 | 19.31 | | 1.23 9 | | |
| Noverl Object Recognition | 57.86 | 39.44 | 16.34 | 11.23 | | 1.31 8 | | |
|  |  |  | | |  | |  |  |

**Table S2:** **Sample size calculation.** Based on our previous studies and pilot experiments, sample size calculation was determined in the online calculating website [**http://powerandsamplesize.com/**](http://powerandsamplesize.com/) **(**Significance level, α= 0.05; Power,1-β= 80%; Matching ratio, κ=1). Formulas and references are as follows:


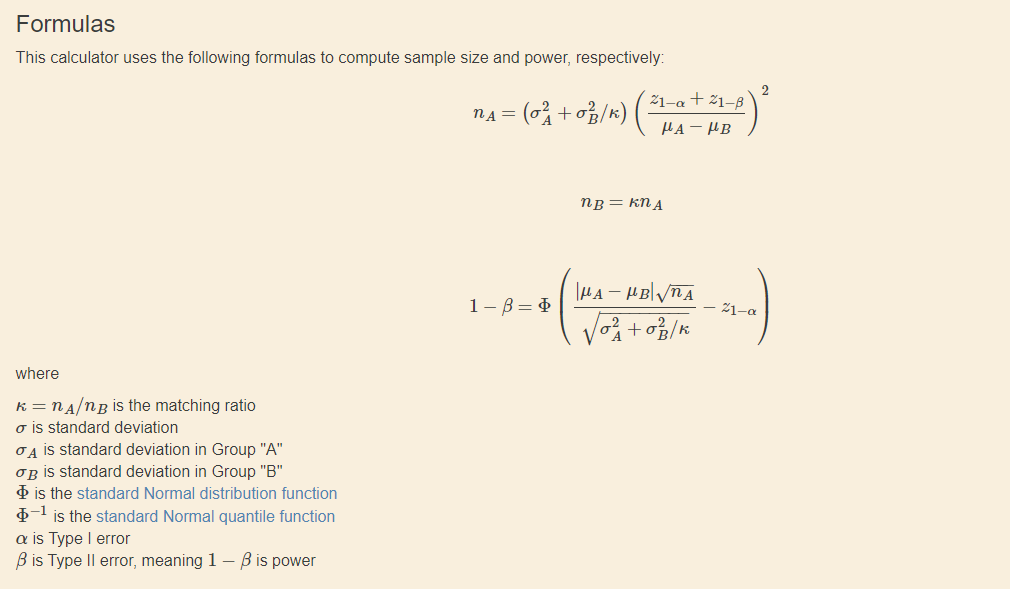


**
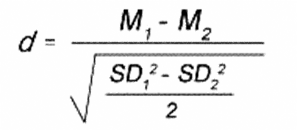
**

**Formulas Reference:**

1. Shein-Chung Chow, Jun Shao, Hansheng Wang, and Yuliya Lokhnygina. Sample Size Calculations in Clinical Research, Third Edition. Taylor & Francis Group, Boca Raton, FL 33487-2742, 2018; pp.48(3.11).

2. Chow S, Shao J, Wang H. 2008. Sample Size Calculations in Clinical Research. 2nd Ed. Chapman & Hall/CRC Biostatistics Series. page 58.
